# Associations between resources and practices of ILD centers and outcomes in patients with idiopathic pulmonary fibrosis: data from the IPF-PRO Registry

**DOI:** 10.1186/s12931-021-01921-7

**Published:** 2022-01-07

**Authors:** Joao A. de Andrade, Tejaswini Kulkarni, Megan L. Neely, Anne S. Hellkamp, Amy Hajari Case, Daniel A. Culver, Kalpalatha Guntupalli, Shaun Bender, Craig S. Conoscenti, Laurie D. Snyder, the IPF-PRO Registry Investigators, Albert Baker, Scott Beegle, John A. Belperio, Rany Condos, Francis Cordova, Daniel A. Culver, Daniel Dilling, John Fitzgerald, Leann Silhan, Kevin R. Flaherty, Kevin Gibson, Mridu Gulati, Kalpalatha Guntupalli, Nishant Gupta, Amy Hajari Case, David Hotchkin, Tristan J. Huie, Robert J. Kaner, Hyun J. Kim, Lisa H. Lancaster, Mark Steele, Joseph A. Lasky, Doug Lee, Timothy Liesching, Randolph Lipchik, Jason Lobo, Tracy R. Luckhardt, Joao A. de Andrade, Yolanda Mageto, Howard Huang, Prema Menon, Yolanda Mageto, Andrew Namen, Justin M. Oldham, Tessy Paul, David Zhang, Anna Podolanczuk, David Lederer, Nina M. Patel, Mary Porteous, Maryl Kreider, Rishi Raj, Paul Mohabir, Murali Ramaswamy, Tonya Russell, Paul Sachs, Zeenat Safdar, Shirin Shafazand, Marilyn Glassberg, Ather Siddiqi, Wael Asi, Barry Sigal, Mary E. Strek, Sally Suliman, Jesse Roman, Jeremy Tabak, Rajat Walia, Timothy P. M. Whelan

**Affiliations:** 1 Vanderbilt University School of Medicine, Nashville, TN USA; 2 University of Alabama at Birmingham, Birmingham, AL USA; 3 Duke Clinical Research Institute, Durham, NC USA; 4 Duke University Medical Center, Durham, NC USA; 5 Piedmont Healthcare, Atlanta, GA USA; 6 Cleveland Clinic, Cleveland, OH USA; 7 Baylor College of Medicine, Houston, TX USA; 8 Boehringer Ingelheim Pharmaceuticals, Inc., Ridgefield, CT USA

**Keywords:** Interstitial lung disease, Pulmonary fibrosis, Health resources, Hospitalization, Tertiary healthcare

## Abstract

**Background:**

Performance benchmarks for the management of idiopathic pulmonary fibrosis (IPF) have not been established. We used data from the IPF-PRO Registry, an observational registry of patients with IPF managed at sites across the US, to examine associations between the characteristics of the enrolling sites and patient outcomes.

**Methods:**

An online survey was used to collect information on the resources, operations, and self-assessment practices of IPF-PRO Registry sites that enrolled ≥ 10 patients. Site variability in 1-year event rates of clinically relevant outcomes, including death, death or lung transplant, and hospitalization, was assessed. Models were adjusted for differences in patient case mix by adjusting for known predictors of each outcome. We assessed whether site-level heterogeneity existed for each patient-level outcome, and if so, we investigated potential drivers of the heterogeneity.

**Results:**

All 27 sites that enrolled ≥ 10 patients returned the questionnaire. Most sites were actively following > 100 patients with IPF (70.4%), had a lung transplant program (66.7%), and had a dedicated ILD nurse leader (77.8%). Substantial heterogeneity was observed in the event rates of clinically relevant outcomes across the sites. After controlling for patient case mix, there were no outcomes for which the site variance component was significantly different from 0, but the p-value for hospitalization was 0.052. Starting/completing an ILD-related quality improvement project in the previous 2 years was associated with a lower risk of hospitalization (HR 0.60 [95% CI 0.44, 0.82]; p = 0.001).

**Conclusions:**

Analyses of data from patients with IPF managed at sites across the US found no site-specific characteristics or practices that were significantly associated with clinically relevant outcomes after adjusting for patient case mix.

*Trial registration* ClinicalTrials.gov, NCT01915511. Registered 5 August 2013, https://clinicaltrials.gov/ct2/show/NCT01915511

**Supplementary Information:**

The online version contains supplementary material available at 10.1186/s12931-021-01921-7.

## Introduction

Idiopathic pulmonary fibrosis (IPF) is a progressive interstitial lung disease (ILD) associated with decline in lung function and high mortality [1]. Although international guidelines for the diagnosis and management of IPF have been published by the American Thoracic Society (ATS), European Respiratory Society (ERS), Japanese Respiratory Society (JRS), and Latin American Thoracic Association (ALAT) [1–4], performance benchmarks for centers diagnosing and managing these patients have not been established. A retrospective study conducted at a single US center found that greater adherence to a bundle of care based on the 2011 ATS/ERS/JRS/ALAT guidelines was associated with improved transplant-free survival after adjustment for age and forced vital capacity (FVC) at baseline [5]. However, there remain few data on how site-specific management practices and resources relate to outcomes in patients with IPF. We hypothesized that the resources, procedures, or organizational characteristics of the sites at which patients with IPF are managed may influence outcomes.

The IPF-PRO Registry (NCT01915511) is an observational multi-center US registry that has enrolled patients with IPF at pulmonary clinics across the US [6]. Registries provide an opportunity to compare practices and outcomes across enrolling centers to generate evidence that may improve clinical care. Previous analyses of data from the IPF-PRO Registry identified a number of patient characteristics that were associated with death or lung transplant [7, 8]. We used data from this registry to describe the enrolling centers’ characteristics and to assess associations between these characteristics and patient outcomes.

## Methods

The design of the IPF-PRO Registry has been published [6]. Briefly, patients with IPF that was diagnosed or confirmed at the enrolling center in the past 6 months were eligible for enrollment. Patients who were participating in a randomized clinical trial or listed for lung transplant could not be enrolled, but patients could participate in a clinical trial or be listed for lung transplant after enrollment. A total of 1002 patients were enrolled at 46 sites (as listed in the “Acknowledgments”) between June 2014 and October 2018. For this analysis, data were extracted from the database in March 2020.

An online survey was used to gather information on the resources, operations, and self-assessment practices of all sites that had enrolled ≥ 10 patients. The survey was developed by adapting the framework used by the US Cystic Fibrosis Foundation clinical benchmarking project [9]. The threshold of 10 patients was selected to ensure that reliable site-level estimates were obtained. Sites completed the questionnaire between 5 February and 1 June 2020, and were instructed to report on their practices and resources prior to the COVID-19 pandemic. Responses based on continuous variables are presented as median (25th percentile, 75th percentile) and categorical variables are presented as number and percentage of sites. The characteristics of patients enrolled at sites included versus not included in this analysis are presented descriptively.

For every site included in the analysis, we estimated the 1-year event rate of the following outcomes from the time of enrollment: (i) death or lung transplant; (ii) hospitalization; (iii) decline in FVC ≥ 10% (mL or % predicted), decline in diffusing capacity of the lungs for carbon monoxide (DLco) ≥ 15% (mmol/min/kPa or % predicted), death, or lung transplant; and (iv) worsening in each patient-reported outcome, death, or lung transplant. The patient-reported outcomes used were the St. George’s Respiratory Questionnaire (SGRQ) [10], the Cough and Sputum Assessment Questionnaire (CASA-Q) [11], the 12-item short-form survey (SF-12) [12] and the EuroQoL 5-D (EQ-5D) index score and visual analog scale (VAS) [13]. The thresholds for worsening of the patient-reported outcomes, i.e. an increase in SGRQ total score ≥ 7, increase in SGRQ activity score ≥ 5, increase in SGRQ impact score ≥ 7, increase in SGRQ symptoms score ≥ 8, decrease in CASA-Q cough domains ≥ 11, decrease in SF-12 mental component summary (MCS) score ≥ 6, decrease in SF-12 physical component summary (PCS) score ≥ 5, decrease in EQ-5D index score ≥ 0.06 and decrease in EQ-5D VAS ≥ 8, were based on published estimates for minimal clinically important differences [14–18].

Site-specific event rates were estimated using the Kaplan–Meier method for all outcomes except hospitalization, for which the cumulative incidence function was used. Site variability in event risk was assessed by fitting a Cox proportional hazards model with a random baseline hazard for each site. Baseline hazard can be interpreted as the relative hazard of a patient at a given site meeting the endpoint relative to a patient at another randomly selected site. The models were adjusted for differences in patient case mix by adjusting for known predictors of each outcome. These predictors were identified through modeling of data from all patients in the IPF-PRO Registry (see Additional file 1: Appendix S1 for details). Age, body mass index (BMI), forced expiratory volume in 1 s (FEV_1_) % predicted, FVC % predicted, DLco % predicted, oxygen use with activity, oxygen use at rest, coronary artery disease or heart failure at enrollment, and diagnosis of IPF prior to referral to the enrolling center, were included as adjustment variables in the model assessing death or lung transplant. BMI, FEV_1_% predicted and oxygen use at rest at enrollment were included as adjustment variables in the model assessing hospitalization. The presence of clinically significant emphysema on HRCT in the opinion of the investigator at enrollment was included as an adjustment variable in the model assessing decline in FVC, death, or lung transplant. Sex, distance to the enrolling site and hospitalization prior to enrollment were included as adjustment variables in the model assessing decline in DLco, death, or lung transplant. Sex and the value of the respective patient-reported outcome at enrollment were included as adjustment variables in the models assessing worsening in a patient-reported outcome, death, or lung transplant.

To generate a valid model fit, sites that enrolled < 25 patients were grouped. To assess whether site-level heterogeneity existed for each patient-level outcome, we tested whether the variance of a random site effect was > 0. If there was evidence of site-level heterogeneity, i.e. if the random site effect was > 0, we investigated potential drivers of the heterogeneity by looking at associations between site practices and outcomes using forward stepwise selection (with an alpha-to-stay of 0.05). That is, covariates were selected one-by-one based on the covariate with the smallest p-value. At each step, the model was adjusted for covariates selected in previous steps, and the selection process terminated when the p-values of all remaining covariates were > 0.05. In the Cox proportional hazards models, missing data were handled using multiple imputation. The Full Conditional Specification method was used to fill in the missing data five times to generate five complete data sets. Each complete data set was analyzed using standard statistical analyses and the results were averaged to generate the final inferential results. All analyses were conducted using SAS version 9.4 or higher (SAS Institute, Cary, NC).

## Results

**Fig. 1:**
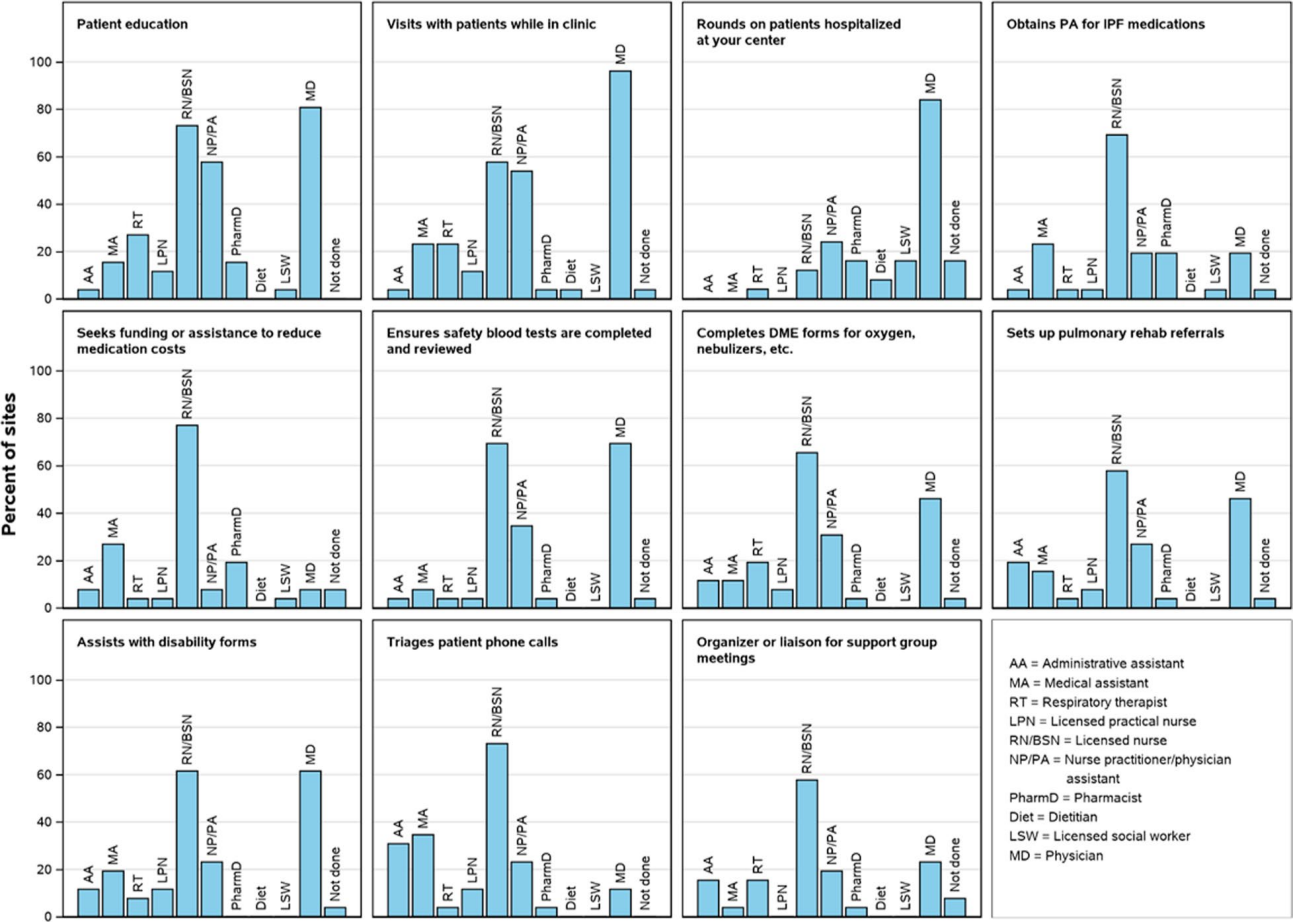
Site staffing models. Based on data from 28 sites. Data were missing from one site for "Rounds on patients hospitalized at your center". *DME* durable medical equipment, *PA* prior authorization

All 27 sites that enrolled ≥ 10 patients and were sent the questionnaire completed it. Twenty-one of these 27 sites were considered academic sites (with a medical school). One site did not complete the section on staffing models. The responses are presented in Table 1. The majority of sites were actively following > 100 patients with IPF (70.4%) and had a lung transplant program at the site (66.7%). Most sites had a dedicated ILD nurse leader to coordinate clinical activities (77.8%), access to a chest radiologist (96.3%) and access to a lung pathologist (100%). The median number of ILD physician specialists was 6. Almost half of the sites (48.1%) held weekly multi-disciplinary conferences to discuss patients. Physicians, nurses and nurse practitioners/physician assistants were the most frequent providers seeing patients in the clinic (Fig. 1). Patients routinely participated in some form of remote monitoring (telehealth, remote pulmonary function tests, electronic medical records held at center, or other forms of remote monitoring) at 22.2% of the sites. An ILD-related quality improvement project had been started or completed in the last 2 years at 40.7% of the sites.

**Table 1:** Responses to questionnaire from sites (n = 27)

Number of enrolled patients	26 (19, 45)
Approximate number of patients with IPF actively followed	
< 25	0
25–50	1 (3.7)
51–100	7 (25.9)
> 100	19 (70.4)
Approximate number of new patient appointments offered each week	
0–5	3 (11.1)
6–10	9 (33.3)
11–15	7 (25.9)
16–20	4 (14.8)
> 20	4 (14.8)
Number of ILD physician specialists at center (full- or part-time)	6 (3, 8)
Dedicated ILD nurse leader to coordinate clinical activities	21 (77.8)
Dedicated ILD nurse practitioner or physician assistant that independently sees patients with ILD	11 (40.7)
Patient calls handled by an ILD registered nurse or nurse practitioner	17 (63.0)
Most patients managed	
By the enrolling site	18 (66.7)
Co-management with community pulmonologist	9 (33.3)
By community pulmonologist primarily	0
Patients routinely participate in some form of remote monitoring	6 (22.2)
Telehealth	3 (11.1)
Remote pulmonary function test monitoring	0
Electronic medical record system-based program at center	1 (3.7)
Other^a^	2 (7.4)
Patients routinely self-monitor their lung function (spirogram) at home	2 (7.4)
Time within which a patient with acute concern/deterioration can typically be seen	
Same/next day if necessary	20 (74.1)
3 days	1 (3.7)
1 week	3 (11.1)
1–2 weeks	1 (3.7)
Other^b^	2 (7.4)
Patient management	
Each individual physician follows his/her own panel of patients	20 (74.1)
Team-based clinic (no assigned patients to a provider)	2 (7.4)
Hybrid model (e.g., individual patients assigned to specific physician but person on call handles all urgent calls)	5 (18.5)
Frequency of multidisciplinary conference to discuss patients	
Weekly	13 (48.1)
Every other week (twice a month)	5 (18.5)
Monthly	7 (25.9)
Quarterly	0
Never	2 (7.4)
Format of MDD	
In person, all participants in same room	20 (74.1)
Remote, by conference call	1 (3.7)
Hybrid, some in room together and others call in	4 (14.8)
Access to chest radiologist (on site or at associated facility)	26 (96.3)
Access to lung pathologist (on site or at associated facility)	27 (100)
Pre-clinic meetings or care planning meetings	
Regular scheduled meetings	3 (11.1)
As-needed meetings	6 (22.2)
No meetings	18 (66.7)
Routinely provide patients with graphs of their lung function while in clinic	13 (48.1)
Center has support group or refers patients to outside support group	25 (92.6)
Support group meets	
Weekly	1 (3.7)
Every other week	0
Monthly	17 (63.0)
Quarterly	6 (22.2)
Twice a year	1 (3.7)
Team member assigned to patient education	10 (37.0)
Routinely provides educational materials in clinic	20 (74.1)
Routinely refers patients to educational websites	23 (85.2)
Educational program/activity dedicated to patients and caregivers at least once a year	17 (63.0)
Local registry/database used for research or quality improvement	18 (66.7)
Started or completed an ILD-related quality improvement project in last 2 years	11 (40.7)
Outcomes self-assessment process in place	2 (7.4)
National Institutes of Health (NIH)-funded research in last 2 years (anyone at center or on team)	18 (66.7)
Center is a member of the Pulmonary Fibrosis Foundation Care Center Network	24 (88.9)
Institution has a lung transplant program	18 (66.7)
Written care protocols/clinical pathways for drug safety monitoring	13 (50.0)

Data are median (25th percentile, 75th percentile) or n (%) of sites. Three centers did not provide data on the number of ILD physician specialists; one center did not provide data on written care protocols

^a^Other responses: “Will likely be rolling out more telehealth in the next 1–2 years”, “Did not do this until the COVID epidemic; now telehealth is big in our program; and will likely remain so”

^b^Other responses: “First 3 days of week, within 1 day, otherwise several days”, “We usually send to the emergency department for serious issues”

The sites that enrolled ≥ 10 patients provided 920 (91.8%) of the 1002 patients in the registry. The median (25th percentile, 75th percentile) number of patients enrolled across the 27 sites was 26 (19, 45). Age, FVC, DLco and oxygen use were similar in these patients compared with patients enrolled at sites not included in the analysis (Additional file 1: Table S1).

### Site-specific event rates

Substantial heterogeneity in site-specific event rates was observed (Table 2). The median (Q1, Q3) site-specific 1-year event rates were 9.8% (6.3%, 17.5%) for death or lung transplant and 21.3% (12.5%, 30.8%) for hospitalization. Similar heterogeneity was observed for the declines in lung function and the composite outcomes that included deterioration in patient-reported outcomes (Table 2).

**Table 2:** Site-specific event rates of outcomes at 1 year

	Median (Q1, Q3) 1-year event rate^a^, %
Death or lung transplant	9.8 (6.3, 17.5)
Hospitalization	21.3 (12.5, 30.8)
Decline in FVC ≥ 10% (mL) or death or lung transplant	32.0 (21.4, 37.5)
Decline in FVC ≥ 10% predicted or death or lung transplant	21.4 (13.9, 28.5)
Decline in DLco ≥ 15% (mmol/min/kPa) or death or lung transplant	35.1 (26.7, 45.7)
Decline in DLco ≥ 15% predicted or death or lung transplant	15.1 (11.1, 25.0)
Increase in SGRQ total score ≥ 7 or death or lung transplant	35.3 (29.8, 43.6)
Increase in SGRQ activity score ≥ 5 or death or lung transplant	48.6 (39.2, 57.5)
Increase in SGRQ impact score ≥ 7 or death or lung transplant	34.8 (30.6, 43.5)
Increase in SGRQ symptoms score ≥ 8 or death or lung transplant	35.7 (25.8, 42.5)
Decrease in CASA-Q cough impact domain ≥ 11 or death or lung transplant	32.3 (26.3, 39.7)
Decrease in CASA-Q cough symptoms domain ≥ 11 or death or lung transplant	31.4 (25.9, 36.3)
Decrease in EuroQoL-5D index score ≥ 0.06 or death or lung transplant	37.7 (28.6, 43.8)
Decrease in EuroQoL-5D VAS score ≥ 8 or death or lung transplant	38.7 (29.5, 45.8)
Decrease in SF-12 MCS score ≥ 6 or death or lung transplant	28.7 (24.0, 33.5)
Decrease in SF-12 PCS score ≥ 5 or death or lung transplant	33.5 (30.3, 40.2)

*CASA-Q* cough and sputum assessment questionnaire, *DLco* diffusing capacity of the lungs for carbon monoxide, *FVC* forced vital capacity, *MCS* mental component summary, *PCS* physical component summary, *SF-12* 12-item short-form survey, *SGRQ* St. George’s Respiratory Questionnaire, *VAS* visual analog scale

^a^Cumulative incidence rate for hospitalization; Kaplan–Meier rates reported for all other outcomes

### Association between site practices and patient outcomes

Twelve sites enrolled between 10 and 25 patients and were grouped in the models. This accounted for 22.2% of the cohort (204 patients). The responses to the questionnaire from sites that enrolled < 25 versus ≥ 25 patients are presented in Additional file 1: Table S2. There were no outcomes for which the site variance component was significantly different from 0, i.e., there was no significant (p < 0.05) site-level heterogeneity for any outcome after controlling for patient case mix, but the p-value for hospitalization was 0.052 (Table 3). When the relationship between site practices and risk of hospitalization was assessed, “starting/completing an ILD-related quality improvement project in the previous 2 years” was associated with a lower risk of hospitalization (HR 0.60 [95% CI 0.44, 0.82]; p = 0.001) and “patients routinely participate in some form of remote monitoring” was associated with a higher risk of hospitalization (HR 1.46 [95% CI 1.04, 2.05]; p = 0.028). After controlling for patient case mix and these site practices, there was no significant site-level heterogeneity for hospitalization (p = 0.17). The responses to the questionnaire from sites with versus without an ILD-related quality improvement project are presented in Additional file 1: Table S3.

**Table 3:** Site variability in outcomes (adjusted for differences in patient case mix)

	Median (Q1, Q3) site-level baseline hazard estimate	p-value for non-zero variance
Death or lung transplant	0.98 (0.92, 1.11)	0.28
Hospitalization	1.06 (0.77, 1.30)	0.052
Decline in FVC ≥ 10% (mL) or death or lung transplant	0.99 (0.94, 1.03)	0.29
Decline in FVC ≥ 10% predicted or death or lung transplant	1.00 (0.97, 1.02)	0.52
Decline in DLco ≥ 15% (mmol/min/kPa) or death or lung transplant	0.97 (0.94, 1.07)	0.11
Decline in DLco ≥ 15% predicted or death or lung transplant	0.99 (0.90, 1.13)	0.12
Increase in SGRQ total score ≥ 7 or death or lung transplant	0.99 (0.98, 1.01)	0.48
Increase in SGRQ activity score ≥ 5 or death or lung transplant	0.99 (0.97, 1.03)	0.40
Increase in SGRQ impact score ≥ 7 or death or lung transplant	0.99 (0.97, 1.01)	0.40
Increase in SGRQ symptoms score ≥ 8 or death or lung transplant	0.99 (0.91, 1.02)	0.20
Decrease in CASA-Q cough impact domain ≥ 11 or death or lung transplant	0.99 (0.94, 1.05)	0.23
Decrease in CASA-Q cough symptoms domain ≥ 11 or death or lung transplant	0.98 (0.91, 1.06)	0.13
Decrease in EuroQoL-5D index score ≥ 0.06 or death or lung transplant	1.00 (0.98, 1.01)	0.72
Decrease in EuroQoL-5D VAS score ≥ 8 or death or lung transplant	1.00 (0.97, 1.03)	0.60
Decrease in SF-12 MCS score ≥ 6 or death or lung transplant	0.99 (0.95, 1.05)	0.32
Decrease in SF-12 PCS score ≥ 5 or death or lung transplant	0.99 (0.98, 1.01)	0.54

## Discussion

We used data from the IPF-PRO Registry to investigate potential associations between the resources and practices of centers with experience in the diagnosis and management of ILD and outcomes in US patients with IPF. To our knowledge, these are the first data investigating such associations. Substantial heterogeneity was observed in the event rates of clinically relevant outcomes across the sites. However, after controlling for differences in known predictors of the outcomes, there were no outcomes for which significant site-level heterogeneity existed i.e., there was no evidence that differences in site practices contributed to the heterogeneity in outcomes across the sites. It remains possible that site-level heterogeneity in outcomes would have been observed if sites with greater differences in resources and practices, e.g. larger academic or regional referral expert centers versus smaller community practices, were compared.

Previous studies have suggested that delayed access to a tertiary care center is associated with worse outcomes in patients with IPF or other ILDs. A prospective study of 129 patients with IPF evaluated at a US tertiary care center found that a greater time from onset of dyspnea to evaluation at the tertiary care center was associated with an increased risk of mortality, after adjustment for age, sex, FVC, payer, and educational attainment [19]. Among 247 patients with IPF from two specialist ILD centers in the UK, patients who were reviewed at a specialist center within 12 months of referral had higher FVC and lower mortality than patients who waited longer to be reviewed [20]. Three-year survival was significantly higher among 144 patients with non-IPF ILDs who received care at a specialized ILD clinic supported by a multidisciplinary team than in a historical cohort of 127 patients whose care was provided by pulmonologists without subspecialty training in ILD or at general clinics without specialized nursing support [21]. The implementation of a multidisciplinary care model at a Canadian clinic was associated with a reduction in the rate of respiratory-related hospitalizations among patients with IPF [22].

Almost half of the IPF-PRO Registry sites that we surveyed had started or completed an ILD-related quality improvement project in the previous 2 years. Quality improvement projects are designed to improve the safety, effectiveness and experience of patient care by enhancing healthcare system performance [23, 24]. We found that patients at sites that had undertaken an ILD-related quality improvement project had a numerically lower risk of hospitalization. The reasons for this finding are unclear, as the changes that sites made to their practices as a result of participating in the quality improvement project are unknown and there were no site practices that were clearly associated with better outcomes after controlling for differences in patient case mix.

Surprisingly, patients from sites where patients participated in routine remote monitoring had a numerically higher risk of hospitalization. The reason for this observation is unknown. By providing more frequent measurements of lung function, monitoring of FVC through home spirometry may enable earlier detection of disease progression [25], but the evidence to support this is limited. While it is possible that patients who participated in remote monitoring had disease progression detected earlier and so were more likely to be hospitalized, it seems unlikely that patients with IPF who had a deterioration that required hospitalization would not have been hospitalized in the absence of remote monitoring. It is possible that in-person visits associate with additional assessments (e.g., chest CT) that may improve clinicians’ comfort not to hospitalize symptomatic patients, or allow earlier intervention that prevents hospitalization.

Strengths of our analyses include the use of a large cohort of patients with IPF enrolled using broad inclusion criteria at multiple sites across the US and adjustment for known predictors of the outcomes. Our analyses also have some limitations. The survey was developed by the research team using the framework of a benchmarking project for a different patient population; however, our survey is aligned with a recently published Delphi survey on the essential components of a specialized ILD clinic [26]. Sites in the IPF-PRO Registry are, in general, regional or national referral centers, which are not representative of all centers at which patients with IPF are managed in the US. It is possible that a study that included more community-based sites as well as referral centers, and so included a greater variety of sites, may have detected associations between site practices and outcomes. The questionnaire used in this study did not collect details of site practices such as quality improvement or palliative care programs. Data on site-specific follow-up and prescription practices were not collected. The use of antifibrotic drugs across the enrolling centers was not assessed in this study, but has previously been shown to be variable across sites in this registry [27]. The association between antifibrotic drug use and outcomes will be the subject of future work. The patient-reported outcome measures used to assess changes in quality of life in the IPF-PRO Registry were not developed in patients with IPF and have limitations as measures of short-term change. Our analyses were based on site practices prior to the COVID-19 pandemic, which resulted in changes in the use of telemedicine that are unlikely to be entirely reversed.

## Conclusions

This analysis of data from over 900 patients with IPF managed at sites with experience in the diagnosis and management of ILD across the US found no site-specific characteristics or practices that were significantly associated with clinically relevant outcomes after adjusting for factors known to be associated with these outcomes. Further studies are needed on resources, systems and management practices that may improve outcomes in patients with IPF.

A podcast of Joao de Andrade and Tejaswini Kulkarni discussing these data is available at: https://www.usscicomms.com/respiratory/deAndrade/IPF-PROsitepractices

## Supplementary Information

**Additional file 1: Appendix S1. **Identification of predictors of clinical outcomes used in adjustment of models. **Table S1.** Baseline characteristics of patients enrolled at sites included versus not included in the analysis. **Table S2.** Responses to questionnaire from sites that enrolled < 25 versus ≥ 25 patients. **Table S3.** Responses to questionnaire from sites with versus without an ILD-related quality improvement project.

## Data Availability

The datasets analyzed during the current study are not publicly available, but are available from the corresponding author on reasonable request.

## Abbreviations

ALAT: Latin American Thoracic Association
ATS: American Thoracic Society
BMI: Body mass index
CASA-Q: Cough and Sputum Assessment Questionnaire
DLco: Diffusing capacity of the lungs for carbon monoxide
ERS: European Respiratory Society
EQ-5D: EuroQoL 5-D
FEV_1_: Forced expiratory volume in 1 s
FVC: Forced vital capacity
HR: Hazard ratio
ILD: Interstitial lung disease
IPF: Idiopathic pulmonary fibrosis
JRS: Japanese Respiratory Society
MCS: Mental component summary
PCS: Physical component summary
SF-12: 12-item short-form survey
SGRQ: St. George’s Respiratory Questionnaire
VAS: Visual analog scale
