# Supplementary material for: Associations between resources and practices of ILD centers and outcomes in patients with idiopathic pulmonary fibrosis: data from the IPF-PRO Registry
Source: Respir Res. 2022 Jan 7;23:3. doi: 10.1186/s12931-021-01921-7 (PMC8740873; doi:10.1186/s12931-021-01921-7)
Supplement: Supplementary file 1 — Additional file 1: Appendix S1. Identification of predictors of clinical outcomes used in adjustment of models. Table S1. Baseline characteristics of patients enrolled at sites included versus not included in the analysis. Table S2. Responses to questionnaire from sites that enrolled < 25 versus ≥ 25 patients. Table S3. Responses to questionnaire from sites with versus without an ILD-related quality improvement project. [file 12931_2021_1921_MOESM1_ESM.docx]

**Additional file**

**Associations between resources and practices of ILD centers and outcomes in patients with idiopathic pulmonary fibrosis: data from the IPF-PRO Registry**

Joao A de Andrade, Tejaswini Kulkarni, Megan L Neely, Anne S Hellkamp, Amy Hajari Case, Daniel A Culver, Kalpalatha Guntupalli, Shaun Bender, Craig S Conoscenti, Laurie D Snyder, on behalf of the IPF-PRO Registry investigators

**Appendix S1:** Identification of predictors of clinical outcomes used in adjustment of models

Candidate predictors for adjustment models were identified based on clinical expertise, review of the literature, and the number of events in the IPF-PRO Registry cohort (*i.e.,* at least 5 events per predictor were required). An initial set of 21 candidate predictors, all measured at the time of enrollment, was identified, comprising demographics (age, sex, insurance type, distance to enrolling center); IPF history and severity (new diagnosis of IPF at enrolling center versus confirmed diagnosis of IPF at enrolling center, diagnostic criteria for IPF [definite versus probable or possible according to 2011 ATS/ERS/JRS/ALAT guidelines], time from symptom onset to new or confirmed diagnosis of IPF at enrolling center, forced expiratory volume in 1 second [FEV_1_] % predicted, forced vital capacity [FVC] % predicted, diffusing capacity of the lungs for carbon monoxide [DLco] % predicted, supplemental oxygen use with activity, supplemental oxygen use at rest); comorbidities and other risk factors (body mass index, cardiac disease, pulmonary hypertension, clinically significant emphysema on HRCT in the opinion of the investigator, pulmonary embolism or deep vein thrombosis, gastro-esophageal reflux disease, smoking history); and hospitalization history (any hospitalization in the 12 months before enrollment, number of respiratory hospitalizations in the 12 months before enrollment). For each outcome, all covariates were entered into the Cox proportional hazards model and backwards selection applied using an alpha-to-stay criterion of 0.05. That is, covariates were removed one-by-one based on the covariate with the largest p-value. The selection process began by including all candidate covariates in a multivariable model and terminated when the p-values of all remaining covariates were ≤0.05. For the adjustment of composite outcomes, predictors of all components were used, *e.g.,* for the model assessing death or lung transplant, all predictors of death and all predictors of lung transplant were included.

**Table S1.** Baseline characteristics of patients enrolled at sites included versus not included in the analysis

|  | **Included in analysis (n=920)** | **Not included in analysis (n=82)** |
| --- | --- | --- |
| Age, years | 70 (65, 75) | 70 (65, 76) |
| Male | 691 (75.1) | 57 (69.5) |
| White | 858 (95.7) | 71 (86.6) |
| Private insurance | 566 (61.5) | 39 (47.6) |
| Region of US |  |  |
| Northeast | 146 (15.9) | 14 (17.3) |
| Midwest | 190 (20.7) | 18 (22.2) |
| South | 509 (55.4) | 35 (43.2) |
| West | 74 (8.1) | 13 (16.0) |
| US territory | 0 | 1 (1.2) |
| Lives in rural area | 187 (20.3) | 6 (7.3) |
| Distance to enrolling center, miles | 36 (14, 100) | 17 (7, 42) |
| Current or former smoker | 620 (67.4) | 50 (61.0) |
| ILD in first degree relative | 171 (19.5) | 15 (20.5) |
| Hospitalization in 12 months prior to enrolment | 248 (28.1) | 24 (30.4) |
| Respiratory hospitalization in 12 months prior to enrolment | 156 (17.7) | 12 (15.2) |
| Referred by pulmonologist | 538 (58.8) | 31 (37.8) |
| Diagnosis of IPF before referral to enrolling center | 412 (44.9) | 34 (41.5) |
| Time from symptom onset to confirmed diagnosis of IPF, months | 15.0 (6.7, 32.4) | 12.0 (6.4, 26.0) |
| Diagnostic criteria for IPF* |  |  |
| Definite | 603 (65.5) | 52 (63.4) |
| Probable | 229 (24.9) | 24 (29.3) |
| Possible | 88 (9.6) | 6 (7.3) |
| FVC % predicted | 69.7 (59.8, 80.2) | 70.9 (53.4, 81.8) |
| FEV_1_ % predicted | 77.1 (65.6, 88.9) | 78.2 (56.5, 94.5) |
| GAP stage^†^ |  |  |
| I | 227 (29.5) | 17 (32.1) |
| II | 411 (53.4) | 26 (49.1) |
| III | 131 (17.0) | 10 (18.9) |
| CPI^‡^ | 52.5 (45.2, 59.8) | 53.0 (42.7, 59.0) |
| DLco % predicted | 42.1 (32.6, 51.4) | 44.3 (33.8, 52.5) |
| Oxygen use with activity | 304 (34.0) | 29 (36.3) |
| Oxygen use at rest | 176 (19.7) | 19 (23.5) |
| Proton pump inhibitor use | 464 (55.7) | 38 (51.4) |
| H2 blocker use | 87 (10.4) | 9 (12.2) |
| Oral steroid use | 107 (12.9) | 9 (12.2) |
| Bronchodilator use | 253 (30.4) | 33 (44.6) |
| Pulmonary vasodilator use | 23 (2.8) | 4 (5.4) |
| Pirfenidone use | 289 (31.4) | 14 (17.1) |
| Nintedanib use | 208 (22.6) | 31 (37.8) |
| SGRQ total score | 39.5 (25.2, 53.7) | 41.3 (27.1, 53.5) |
| SF-12 mental component score | 54.1 (45.8, 59.2) | 51.9 (42.4, 58.6) |
| SF-12 physical component score | 38.9 (31.1, 46.0) | 38.0 (32.4, 45.8) |
| CASA-Q cough symptoms domain | 58.3 (41.7, 75.0) | 66.7 (41.7, 75.0) |
| CASA-Q cough impact domain | 78.1 (56.3, 93.8) | 75.0 (56.3, 93.8) |
| EuroQoL score | 0.8 (0.7, 1.0) | 0.8 (0.6, 0.9) |
| EuroQoL visual analog scale | 75 (60, 85) | 70 (50, 85) |
| History of coronary artery disease | 266 (29.0) | 31 (37.8) |
| History of congestive heart failure | 53 (5.8) | 4 (4.9) |
| History of atrial fibrillation or flutter | 91 (9.9) | 11 (13.4) |
| History of stroke or intracerebral hemorrhage | 35 (4.9) | 3 (3.7) |
| History of deep vein thrombosis or pulmonary embolism | 33 (3.6) | 9 (11.0) |
| History of gastroesophageal reflux disease | 519 (56.7) | 41 (50.0) |
| History of sleep apnea | 260 (28.4) | 17 (20.7) |
| History of chronic kidney disease | 31 (3.4) | 1 (1.2) |
| History of cirrhosis or chronic liver disease | 16 (1.7) | 4 (4.9) |
| History of lung cancer | 3 (0.3) | 0 |
| History of diabetes | 191 (20.8) | 14 (17.1) |
| History of pulmonary hypertension | 62 (6.8) | 9 (11.0) |

Data are median (25^th^ percentile, 75^th^ percentile) or n (%) of patients. *According to 2011 ATS/ERS/JRS/ALAT diagnostic guidelines [1]. ^†^Gender, age, lung physiology (GAP) staging system [2]. ^‡^Composite physiologic index [3].

**Table S2**. Responses to questionnaire from sites that enrolled <25 versus ≥25 patients

|  | Sites enrolled <25 patients (n=12) | Sites enrolled ≥25 patients (n=15) |
| --- | --- | --- |
| Number of enrolled patients | 18 (14, 19) | 40 (27, 52) |
| Approximate number of patients with IPF actively followed |  |  |
| <25 | 0 | 0 |
| 25–50 | 1 (8.3) | 0 |
| 51–100 | 5 (41.7) | 2 (13.3) |
| >100 | 6 (50.0) | 13 (86.7) |
| Approximate number of new patient appointments offered each week |  |  |
| 0–5 | 2 (16.7) | 1 (6.7) |
| 6–10 | 3 (25.0) | 6 (40.0) |
| 11–15 | 4 (33.3) | 3 (20.0) |
| 16–20 | 2 (16.7) | 2 (13.3) |
| >20 | 1 (8.3) | 12 (80.0) |
| Number of ILD physician specialists at center (full- or part-time) | 3 (2, 5) | 7 (6, 8) |
| Dedicated ILD nurse leader to coordinate clinical activities | 9 (75.0) | 12 (80.0) |
| Dedicated ILD nurse practitioner or physician assistant that independently sees patients with ILD | 4 (33.3) | 7 (46.7) |
| Patient calls handled by an ILD registered nurse or nurse practitioner | 7 (58.3) | 10 (66.7) |
| Most patients managed: |  |  |
| By the enrolling site | 10 (83.3) | 8 (53.3) |
| Co-management with community pulmonologist | 2 (16.7) | 7 (46.7) |
| By community pulmonologist primarily | 0 | 0 |
| Patients routinely participate in some form of remote monitoring | 4 (33.3) | 2 (13.3) |
| Telehealth | 2 (16.7) | 1 (6.7) |
| Remote pulmonary function test monitoring | 0 | 0 |
| Electronic medical record system-based program at center | 1 (8.3) | 0 |
| Other | 1 (8.3) | 1 (6.7) |
| Patients routinely self-monitor their lung function (spirogram) at home | 2 (16.7) | 0 |
| Time within which a patient with acute concern/deterioration can typically be seen |  |  |
| Same/next day if necessary | 10 (83.3) | 10 (66.7) |
| 3 days | 0 | 1 (6.7) |
| 1 week | 1 (8.3) | 2 (13.3) |
| 1–2 weeks | 0 | 1 (6.7) |
| Other | 1 (8.3) | 1 (6.7) |
| Patient management |  |  |
| Each individual physician follows his/her own panel of patients | 8 (66.7) | 12 (80.0) |
| Team-based clinic (no assigned patients to a provider) | 0 | 2 (13.3) |
| Hybrid model (e.g., individual patients assigned to specific physician but person on call handles all urgent calls) | 4 (33.3) | 1 (6.7) |
| Frequency of multi-disciplinary conference to discuss patients |  |  |
| Weekly | 4 (33.3) | 9 (60.0) |
| Every other week (twice a month) | 3 (25.0) | 2 (13.3) |
| Monthly | 5 (41.7) | 2 (13.3) |
| Quarterly | 0 | 0 |
| Never | 0 | 2 (13.3) |
| Format of MDD |  |  |
| In person, all participants in same room | 11 (91.7) | 9 (60.0) |
| Remote, by conference call | 0 | 1 (6.7) |
| Hybrid, some in room together and others call in | 1 (8.3) | 3 (20.0) |
| Access to chest radiologist (on site or at associated facility) | 11 (91.7) | 15 (100) |
| Access to lung pathologist (on site or at associated facility) | 12 (100) | 15 (100) |
| Pre-clinic meetings or care planning meetings |  |  |
| Regular scheduled meetings | 2 (16.7) | 1 (6.7) |
| As-needed meetings | 5 (41.7) | 1 (6.7) |
| No meetings | 5 (41.7) | 13 (86.7) |
| Routinely provide patients with graphs of their lung function while in clinic | 6 (50.0) | 7 (46.7) |
| Center has support group or refers patients to outside support group | 10 (83.3) | 15 (100) |
| Support group meets: |  |  |
| Weekly | 0 | 1 (6.7) |
| Every other week | 0 | 0 |
| Monthly | 9 (75.0) | 8 (53.3) |
| Quarterly | 1 (8.3) | 5 (33.3) |
| Twice a year | 0 | 1 (6.7) |
| Team member assigned to patient education | 7 (58.3) | 3 (20.0) |
| Routinely provides educational materials in clinic | 12 (100) | 8 (53.3) |
| Routinely refers patients to educational websites | 9 (75.0) | 14 (93.3) |
| Educational program/activity dedicated to patients and caregivers at least once a year | 8 (66.7) | 9 (60.0) |
| Local registry/database used for research or quality improvement | 8 (66.7) | 10 (66.7) |
| Started or completed an ILD-related quality improvement project in last 2 years | 7 (58.3) | 4 (26.7) |
| Outcomes self-assessment process in place | 2 (16.7) | 0 |
| National Institutes of Health (NIH)-funded research in last 2 years (anyone at center or on team) | 3 (25.0) | 15 (100) |
| Center is a member of the Pulmonary Fibrosis Foundation Care Center Network | 9 (75.0) | 15 (100) |
| Institution has a lung transplant program | 5 (41.7) | 13 (86.7) |
| Written care protocols/clinical pathways for drug safety monitoring | 6 (50.0) | 7 (46.7) |

Data are median (25^th^ percentile, 75^th^ percentile) or n (%) of sites.

**Table S3**. Responses to questionnaire from sites with versus without an ILD-related quality improvement project

|  | Sites with ILD-related quality improvement project (n=11) | Sites without ILD-related quality improvement project (n=16) |
| --- | --- | --- |
| Number of enrolled patients | 19 (13, 28) | 33 (21, 47) |
| Approximate number of patients with IPF actively followed |  |  |
| <25 | 0 | 0 |
| 25–50 | 1 (9.1) | 0 |
| 51–100 | 3 (27.3) | 4 (25.0) |
| >100 | 7 (63.6) | 12 (75.0) |
| Approximate number of new patient appointments offered each week |  |  |
| 0–5 | 1 (9.1) | 2 (12.5) |
| 6–10 | 3 (27.3) | 6 (37.5) |
| 11–15 | 3 (27.3) | 4 (25.0) |
| 16–20 | 2 (18.2) | 2 (12.5) |
| >20 | 2 (18.2) | 2 (12.5) |
| Number of ILD physician specialists at center (full- or part-time) | 6 (3, 8) | 6 (3, 8) |
| Dedicated ILD nurse leader to coordinate clinical activities | 10 (90.9) | 11 (68.8) |
| Dedicated ILD nurse practitioner or physician assistant that independently sees patients with ILD | 5 (45.5) | 6 (37.5) |
| Patient calls handled by an ILD registered nurse or nurse practitioner | 9 (81.8) | 8 (50.0) |
| Most patients managed: |  |  |
| By the enrolling site | 6 (54.5) | 12 (75.0) |
| Co-management with community pulmonologist | 5 (45.5) | 4 (25.0) |
| By community pulmonologist primarily | 0 | 0 |
| Patients routinely participate in some form of remote monitoring |  |  |
| Telehealth | 2 (18.2) | 1 (6.3) |
| Remote pulmonary function test monitoring | 0 | 0 |
| Electronic medical record system-based program at center | 0 | 1 (6.3) |
| Other | 1 (9.1) | 1 (6.3) |
| Patients routinely self-monitor their lung function (spirogram) at home | 1 (9.1) | 1 (6.3) |
| Time within which a patient with acute concern/deterioration can typically be seen |  |  |
| Same/next day if necessary | 8 (72.7) | 12 (75.0) |
| 3 days | 0 | 1 (6.3) |
| 1 week | 2 (18.2) | 1 (6.3) |
| 1–2 weeks | 0 | 1 (6.3) |
| Other | 1 (9.1) | 1 (6.3) |
| Patient management |  |  |
| Each individual physician follows his/her own panel of patients | 7 (63.6) | 13 (81.3) |
| Team-based clinic (no assigned patients to a provider) | 1 (9.1) | 1 (6.3) |
| Hybrid model (e.g., individual patients assigned to specific physician but person on call handles all urgent calls) | 3 (27.3) | 2 (12.5) |
| Frequency of multi-disciplinary conference to discuss patients |  |  |
| Weekly | 6 (54.5) | 7 (43.8) |
| Every other week (twice a month) | 2 (18.2) | 3 (18.8) |
| Monthly | 3 (27.3) | 4 (25.0) |
| Quarterly | 0 | 0 |
| Never | 0 | 2 (12.5) |
| Format of MDD |  |  |
| In person, all participants in same room | 9 (81.8) | 11 (68.8) |
| Remote, by conference call | 0 | 1 (6.3) |
| Hybrid, some in room together and others call in | 2 (18.2) | 2 (12.5) |
| Access to chest radiologist (on site or at associated facility) | 11 (100) | 15 (93.8) |
| Access to lung pathologist (on site or at associated facility) | 11 (100) | 16 (100) |
| Pre-clinic meetings or care planning meetings |  |  |
| Regular scheduled meetings | 3 (27.3) | 0 |
| As-needed meetings | 3 (27.3) | 3 (18.8) |
| No meetings | 5 (45.5) | 13 (81.3) |
| Routinely provide patients with graphs of their lung function while in clinic | 7 (63.6) | 6 (37.5) |
| Center has support group or refers patients to outside support group | 10 (90.9) | 15 (93.8) |
| Support group meets: |  |  |
| Weekly | 1 (9.1) | 0 |
| Every other week | 0 | 0 |
| Monthly | 8 (72.7) | 9 (56.3) |
| Quarterly | 1 (9.1) | 5 (31.3) |
| Twice a year | 0 | 1 (6.3) |
| Team member assigned to patient education | 6 (54.5) | 12 (75.0) |
| Routinely provides educational materials in clinic | 9 (81.8) | 11 (68.8) |
| Routinely refers patients to educational websites | 9 (81.8) | 14 (87.5) |
| Educational program/activity dedicated to patients and caregivers at least once a year | 7 (63.6) | 10 (62.5) |
| Local registry/database used for research or quality improvement | 8 (72.7) | 10 (62.5) |
| Started or completed an ILD-related quality improvement project in last 2 years | 11 (100) | 0 |
| Outcomes self-assessment process in place | 2 (18.2) | 0 |
| National Institutes of Health (NIH)-funded research in last 2 years (anyone at center or on team) | 5 (45.5) | 13 (81.3) |
| Center is a member of the Pulmonary Fibrosis Foundation Care Center Network | 8 (72.7) | 16 (100) |
| Institution has a lung transplant program | 7 (63.6) | 11 (68.8) |
| Written care protocols/clinical pathways for drug safety monitoring | 7 (63.6) | 6 (37.5) |

Data are median (25^th^ percentile, 75^th^ percentile) or n (%) of sites.

**References**

1. Raghu G, Collard HR, Egan JJ, et al. An official ATS/ERS/JRS/ALAT statement: idiopathic pulmonary fibrosis: evidence-based guidelines for diagnosis and management. Am J Respir Crit Care Med 2011;183:788–824.
2. Ley B, Ryerson CJ, Vittinghoff E, et al. A multidimensional index and staging system for idiopathic pulmonary fibrosis. Ann Intern Med 2012;156:684–691.
3. Wells AU, Desai SR, Rubens MB, et al. Idiopathic pulmonary fibrosis: a composite physiologic index derived from disease extent observed by computed tomography. Am J Respir Crit Care Med 2003;167:962–969.
